# Do Email and Mobile Phone Prompts Stimulate Primary School Children to Reuse an Internet-Delivered Smoking Prevention Intervention?

**DOI:** 10.2196/jmir.3069

**Published:** 2014-03-18

**Authors:** Henricus-Paul Cremers, Liesbeth Mercken, Rik Crutzen, Paul Willems, Hein de Vries, Anke Oenema

**Affiliations:** ^1^School for Public Health and Primary Care (CAPHRI)Department of Health PromotionMaastricht UniversityMaastrichtNetherlands; ^2^School for Nutrition, Toxicology and Metabolism (NUTRIM)Department of Human Movement SciencesMaastricht UniversityMaastrichtNetherlands

**Keywords:** Internet, Internet-delivered intervention, prompts, primary school children, computer-tailoring

## Abstract

**Background:**

Improving the use (eg, initial visit and revisits) of Internet-delivered interventions to promote healthy lifestyles such as non-smoking is one of the largest challenges in the field of eHealth. Prompts have shown to be effective in stimulating reuse of Internet-delivered interventions among adults and adolescents. However, evidence concerning effectiveness of prompts to promote reuse of a website among children is still scarce.

**Objective:**

The aim of this study is to investigate (1) whether prompts are effective in promoting reuse of an intervention website containing information on smoking prevention for children, (2) whether the content of the prompt is associated with its effect in terms of reuse, and (3) whether there are differences between children who do or do not respond to prompts.

**Methods:**

The sample of this cluster-randomized study consisted of 1124 children (aged 10-11 years) from 108 Dutch primary schools, who were assigned to the experimental group of an Internet-delivered smoking prevention intervention study. All participants completed a Web-based questionnaire on factors related to (non-)smoking. Schools were randomized to a no-prompt group (n=50) or a prompt group (n=58). All children could revisit the intervention website, but only the children in the prompt group received email and SMS prompts to revisit the website. Those prompt messages functioned as a teaser to stimulate reuse of the intervention website. Reuse of the website was objectively tracked by means of a server registration system. Repeated measures analysis of variance and linear regression analysis were performed to assess the effects of prompts on website reuse and to identify individual characteristics of participants who reuse the intervention website.

**Results:**

Children in the prompt group reused the intervention website significantly more often compared to children in the no-prompt group (*B*=1.56, *P*<.001). Prompts announcing new animated videos (*F*_1,1122_=9.33, *P*=.002) and games about (non-)smoking on the website (*F*_1,1122_=8.28, *P*=.004) resulted in most reuse of the website. Within the prompt group, children with a low socioeconomic status (SES) reused the intervention website more often (*B*=2.19, *P*<.001) than children of high SES (*B*=0.93, *P*=.005).

**Conclusions:**

Prompts can stimulate children to reuse an intervention website aimed at smoking prevention. Prompts showed, furthermore, to stimulate children of a low SES slightly more to reuse an intervention website, which is often a difficult target group in terms of stimulating participation. However, the number of revisits was quite low, which requires further study into how prompts can be optimized in terms of content and frequency to improve the number of revisits.

**Trial Registration:**

Netherlands Trial Register Number: NTR3116; http://www.trialregister.nl/trialreg/admin/rctview.asp?TC=3116 (Archived by WebCite at http://www.webcitation.org/6O0wQYuPI).

## Introduction

Smoking prevalence rates among Dutch primary school children increase rapidly when they make the transition to secondary school [1]; therefore, it is important to prevent the uptake of smoking before positive attitudes and beliefs toward smoking are formed [2]. Internet-delivered computer-tailored interventions have the potential to be effective in promoting healthy lifestyle behaviors among adults, adolescents [3-7], and children [8,9]. Internet-delivered interventions also hold the promise of reaching large numbers of people; however, achieving that reach is a problem for all these target groups, including children. Although large numbers of children can be reached when health promotion interventions, such as smoking prevention interventions, are implemented in the school setting [10,11], dropout of children in those interventions is high [12]. Optimal use and reuse of programs is a prerequisite for achieving optimal program impact [13]. To improve use and reuse of healthy lifestyle-promoting Internet-delivered interventions and to prevent premature dropout from such programs, new strategies are required to stimulate reuse of such interventions [14,15] and to remind participants of their involvement in an intervention.

Prior studies have shown that frequent use of an Internet-delivered intervention resulted in higher smoking cessation rates among adults and adolescents [16-19]; it is to be expected that sustained use of an Internet-delivered intervention will also have a positive effect on the smoking behavior of children. However, no research concerning a dose-response relationship in smoking prevention in children is available yet. To stimulate reuse of an Internet-delivered intervention, periodic prompts may be a valuable tool [20]. Previous studies among adults [20-23] and adolescents [24,25] have demonstrated that the provision of prompts had a positive effect on reuse of an intervention website, for example on the number of log-ins. Evidence on how children respond to prompt messages when they are involved in an Internet-delivered intervention is scarce. Therefore, it is important to study the effects of prompts in primary school children to increase the potential reuse of effective interventions.

Reuse of Internet-delivered interventions is dependent on both the intervention characteristics (eg, updates of the intervention website or email contact) and the individual characteristics of the participants [23,26,27]. Furthermore, there is evidence that prompt content may be of importance for intervention use and reuse, even though no conclusive evidence has been found as to what type of content is most effective in stimulating curiosity among participants to reuse an Internet-delivered intervention. Prior research among adults has indicated that participants were more willing to log in to an intervention website if they received prompt messages containing a preview of new information compared to standard prompt messages (a message that reminded people of their previous visit and invited them to reuse the website without addressing new content added to the intervention website) [28]. Furthermore, it is plausible that individual characteristics of participants, such as age, gender, or socioeconomic status (SES), are associated with whether or not they reuse an Internet-delivered intervention [21,26,27].

Prompts can be sent in various ways and the most efficient and low-cost options may be using current technologies [ie, email and short message service (SMS)]. These can be low in cost when compared to conventional postal mail or telephone calls and are relatively easy to implement in Internet-delivered computer-tailored interventions [23,29-31]. Furthermore, the use of multimedia such as the Internet or mobile phones among Dutch children (aged 10-11 years) is relatively high (93% of these children use email and 60-69% have their own mobile phone) [32-35].

The objectives of the present study are to examine (1) whether prompts will stimulate primary school children to reuse a smoking prevention website, (2) whether the prompt content is related to its effect in terms of reuse, and (3) which individual characteristics of children are associated with a higher likelihood to respond to prompts and reuse an intervention website.

## Methods

### Study Design, Participants, and Procedure

The study was conducted as a cluster-randomized controlled trial in which 108 primary schools in the Netherlands were randomized to either a prompt (n=58) or no-prompt group (n=50) of a larger smoking prevention intervention study called “Fun without Smokes” [36]. Both groups had access to the Fun without Smokes website and one group received prompts reminding them to revisit the website (prompt group), while the other group did not receive prompts. Participants in the present study were children in grade 7 (aged 10-11 years). Primary schools were recruited by Municipal Health Promotion Organizations and Maastricht University for participation in the smoking prevention intervention study. Children in grade 7 of all participating schools were included in the intervention study, unless they or their parents refused to be involved (passive informed consent procedure). This study was approved by the Medical Ethics Committee of the Atrium-Orbis-Zuyd Hospital (NL32093.096.11/MEC 11-T-25).

**Figure 1:**
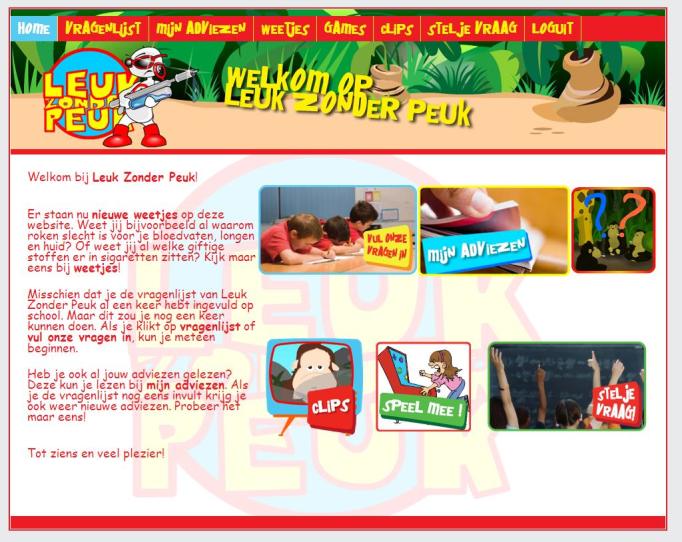
‘Fun without Smokes’ website.

In October 2011, all children received personalized log-in codes (username and password) to access the Fun without Smokes website (Figure 1) and were asked to fill out a Web-based questionnaire at their primary school concerning their smoking status and other factors related to smoking. After completion, children in both the prompt and no-prompt group received personalized computer-tailored feedback letters in their own email box and at the Fun without Smokes website. Those feedback letters were not only tailored to children’s personal characteristics, but also to sociocognitive variables (eg, attitude, social influences, and self-efficacy expectations) toward (non-)smoking. The children in the prompt group received 6 prompt messages to stimulate them to reuse the Fun without Smokes website, where they could read new information concerning (non-)smoking, play games, or watch animated videos with non-smoking content. After children completed the questionnaire at school, they were able to use the Web-based intervention at home.

Use and reuse of the website was monitored by means of server registrations. Data gathered during the first year of the intervention study (October 2011-September 2012) was used in the analyses. Since prompts were sent via email and SMS, inclusion criteria for the present study were that children had entered a complete and verifiable email address or mobile phone number and that they had indicated they actually use this email address or mobile phone number.

### Intervention Website

The Fun without Smokes website was accessible to the children in both the prompt and no-prompt groups during the intervention period. Core elements of the Fun without Smokes website were the Web-based questionnaire and the computer-tailored feedback letters. Furthermore, the website provided information on non-smoking through facts concerning non-smoking, anti-smoking games, and short animated videos with non-smoking content. Furthermore, children had the opportunity to ask questions concerning (non-)smoking. To create a website that was most attractive and appreciated, children from the target group were involved in the development process [36]. It was expected that higher reuse of the website would also increase exposure to the tailored content. For that reason, children were also able to complete the Web-based questionnaire as often as they wanted and receive renewed computer-tailored feedback.

### Email and SMS Prompts

In the computer-tailored feedback letters, it was indicated that participants in both the prompt and no-prompt groups were able to reuse the website during the intervention period. Children who had entered an email and/or mobile phone number in the prompt group received 6 prompt messages within 9 months to stimulate them to reuse the Fun without Smokes website. The prompts were sent via email and/or as SMS messages, depending on whether the child had provided an email account and/or mobile phone number and had indicated to use this device. Children received an email and SMS message if they provided both (ie, email address and phone number), otherwise they received only an email or SMS. Children without email or mobile phone did not receive the prompts.

All prompt messages varied in content and were sent at different time periods. The first 3 prompts were sent 1, 2, and 3 months after the baseline questionnaire was completed. The last 3 prompts were sent 5, 7, and 9 months after baseline. In accordance with the prompts, some of the content of the intervention website was refreshed to address a new topic relevant for smoking prevention. The prompts functioned as a teaser to increase curiosity among the children to view the new content at the Fun without Smokes website (eg, “Hi, now there is a funny game on the Fun without Smokes website. Check it out today and play this game!”). The content of the first and second prompt indicated that new facts on (non-)smoking were posted on the website (Table 1), the third prompt announced that new animated videos, including non-smoking messages, were posted on the website, the fourth prompt reported that a game was available about (non-)smoking, the fifth prompt mentioned new facts about (non-)smoking, and the last prompt announced a new game of non-smoking. Every prompt also included the personal log-in codes of the Fun without Smokes website, to make sure that the children could access the website immediately.

Participants in the no-prompt group also had access to the new information, games, and videos. However, reuse of the Fun without Smokes website was dependent on their own initiative since they did not receive any of the 6 prompt messages. They received their personal log-in codes at the baseline measurement of Fun without Smokes and were asked to save those codes. If they lost the codes, they were able to request them at the Fun without Smokes website.

**Table 1:** Period and content of prompts posted on the website.

Prompt	Prompt period	Prompt content
Prompt 1	1 month after baseline	Facts about (non-)smoking
Prompt 2	2 months after baseline	New facts about (non-)smoking
Prompt 3	3 months after baseline	New animated videos
Prompt 4	5 months after baseline	Game on (non-)smoking
Prompt 5	7 months after baseline	New facts about (non-)smoking
Prompt 6	9 months after baseline	New game on (non-)smoking

### Measurements

#### Overview

Primary outcome measure of the present study is reuse of the Fun without Smokes website. Use and reuse of the website was assessed objectively by means of a server registration system. Reuse was measured as a continuous variable, based on the number of clicks (ranging from 0 to 95). Characteristics of the user and reuser such as age, gender ethnicity, and SES were derived from the baseline questionnaire that the children completed in the classroom on the Fun without Smokes website.

#### Assessment of Website Use and Reuse

Data on website visits was retrieved from a specific server registration system, which made it possible to register website access for each individual child. Using the personal usernames of all participating children, we tracked how often and when (date and time) they reused the Fun without Smokes website.

Reuse of the website was calculated by summing all clicks in the different website components from the first till the last prompt. The clicks in the first month of the intervention period were not included in the calculation since children of both the prompt and no-prompt groups had to complete the Web-based questionnaire at their primary school and no prompt messages were sent in this period. By using this approach, reuse of the intervention website indicates how intensively the website was reused after the prompts were sent.

#### Self-Reported Data Retrieved From the Baseline Questionnaire

Availability of email addresses for the participating children was measured in the Web-based questionnaire. Children were able to fill out their email address (scored as “1”) or indicate if they had no email address or had forgotten their email address (scored as “0”). Children could also indicate whether they actually used their email address (coded 1) or not (coded 0).

Availability of mobile phone numbers for the participants was also measured in the questionnaire. Children having a mobile phone number were scored with a “1”, whereas children without a mobile phone number or if they had forgotten their mobile phone number were scored with a “0”. Children could also indicate whether they actually used their mobile phone (coded 1) or not (coded 0).

In the questionnaire, the following background variables were measured: age (in years), gender (1=boy; 2=girl), ethnicity, and SES of the participants. Ethnicity indicated whether a child had a Western or non-Western background. A child was considered to be of Western ethnic background (coded 1) if he or she and both parents had been born in the Netherlands, another European country, North America, Oceania, Indonesia (a former colony of the Netherlands), or Japan. Otherwise the child was considered to be of non-Western ethnic background (coded 2) [37]. SES was based on their postal code, which the children had provided in the questionnaire. The Netherlands Institute for Social Research (Dutch government agency that conducts research into the social aspects of all areas of government policy) calculated the SES in every 4-digit postal code area based on income, occupation, and education of Dutch inhabitants in 2010 [38]. In this study, low SES was coded with a “0” and a high SES was coded with a “1”.

The data from the server registration system and the data from the baseline questionnaire could be linked by means of the personal usernames, making it possible to unobtrusively observe if a participant reused the intervention website after a prompt message was sent and to combine usage information with individual data of the users.

### Statistical Analyses

General descriptives were carried out to describe the sample under study. Differences at baseline between characteristics of children (ie, age, gender, ethnicity, SES, having/using their email address, and having/using their mobile phone) in the no-prompt and prompt group were analyzed with chi-square and *t* test analyses.

A multiple linear regression analysis was conducted to identify whether there was a difference in website reuse between the prompt and the no-prompt groups. In this analysis, reuse of the intervention website was the dependent variable, and group and demographic characteristics were the independent variables. To identify whether there were differential effects of the prompt condition based on demographic characteristics, a linear regression analysis was done that included group*demographic variable interaction terms (ie, age, gender, ethnicity, or SES). If interaction effects were present, separate analyses were performed for two subgroups of a variable.

To indicate which prompt(s) motivated children most to reuse the Fun without Smokes website, a repeated-measures analysis of variance (ANOVA) was carried out. In this analysis, the number of clicks in the separate prompt periods were analyzed between the prompt and no-prompt group. All analyses were performed in SPSS 20.0. *P* values were said to be significant if they were equal to or lower than .05. Interaction effects were considered to be significant if the *P* value was equal to or lower than .10 to reduce potential type I errors [39].

## Results

### Basic Characteristics

A total of 1124 children met the inclusion criteria and were included in the analyses (13.87%, 181/1305, were excluded). As shown in Table 2, in the total sample, more girls (55.43%, 623/1124) and more children of a Western ethnic background were included (85.50%, 961/1124). Furthermore, fewer children were of high SES (43.06%, 484/1124). None of the differences between the prompt and the no-prompt groups were statistically significant. Most children had an email address and made use of this email address (98.49%, 1107/1124). A minority of the children had and used a mobile phone (15.04%, 169/1124).

### Effect of Prompts on Reusing the Intervention Website

Mean reuse of the intervention website was 2.14 times (SD 7.53) in the prompt group and 0.47 times (SD 2.30) in the no-prompt group and this difference was significant (*B*=1.56, *P*<.001).

### Association between Child Characteristics and Reuse of the Intervention Website

Mean reuse of the intervention website in the prompt group among children of low SES was 3.03 times (SD 9.84) and among high SES children 1.37 times (SD 4.62). Moreover, Table 3 shows that only the “group by SES” interaction term was significant (*B*=−1.22, *P*=.06). Analyses stratified for high and low SES revealed that children of low SES in the prompt group used the website more often (*B*=2.19, *P*<.001) than high SES children in the prompt group (*B*=0.93, *P*=.005). There was no significant difference observed of SES in the no-prompt group.

**Table 2:** Basic characteristics.

Characteristic	Total sample (n=1124)	Prompt (n=586)	No prompt (n=538)	*t*	X^2^	df	*P*
Age in years, mean (SD)	10.35 (0.57)	10.32 (0.56)	10.38 (0.57)	1.62	-	1098	.11
Gender, n (%) girl	623 (55.43)	336 (57.34)	287 (53.35)	-	1.81	1	.18
Ethnicity, n (%) Western	961 (85.50)	500 (85.32)	461 (85.69)	-	0.03	1	.86
SES^a^, n (%) high SES	484 (43.06)	245 (41.81)	239 (44.42)	-	0.13	1	.72
Email address, n (%) yes	1120 (99.64)	585 (99.83)	535 (99.44)	-	1.18	1	.28
Email address use, n (%) yes	1107 (98.49)	577 (98.46)	530 (98.51)	-	0.46	1	.50
Mobile phone, n (%) yes	175 (15.57)	84 (14.33)	91 (16.91)	-	0.14	1	.71
Mobile phone use, n (%) yes)	169 (15.04)	83 (14.16)	86 (15.99)	-	2.44	1	.12

^a^SES: socioeconomic status

**Table 3:** Interaction effects between subgroups and group on reuse of the intervention website^a^.

Group/subgroup	*B*	95% CI	*P*
Age	−1.65	−4.58 to 1.28	.27
Gender (1=male; 2=female)	0.82	−2.50 to 4.13	.63
Ethnicity (1=Western; 2=non-Western)	−0.43	−5.11 to 4.26	.86
SES^b^ (0=low SES; 1=high SES)	2.26	−1.03 to 5.55	.18
Group (0=no prompt; 1=prompt)	−5.76	−17.95 to 6.44	.36
Age*Group	0.77	−0.37 to 1.92	.19
Gender*Group	−0.17	−1.46 to 1.13	.80
Ethnicity*Group	0.16	−1.68 to 1.99	.87
SES*Group	−1.22	−2.50 to 0.07	.06

^a^ *R* ^*2*^=.037

^b^SES: socioeconomic status

### Content of the Prompts

**Figure 2:**
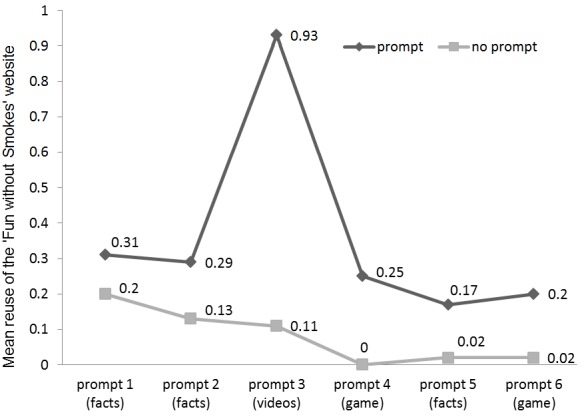
Means of reuse of the "Fun without Smokes" website between prompt and no-prompt group. X-axis time points: prompt 1= 1 month; prompt 2= 2 months; prompt 3= 3 months; prompt 4= 5 months; prompt 5= 7 months; prompt 6= 9 months.

In Figure 2, the mean reuse of the website at all 6 time points is plotted for the prompt and no-prompt groups. Reuse of the website is higher in the prompt group as compared to the no-prompt group after every prompt (*F*_1,1122_=3.66, *P*=.04), with larger differences between the second and third (*F*_1,1122_=9.33, *P*=.002) and between the third and fourth prompt periods (*F*_1,1122_=8.28, *P*=.004). The third prompt announced that new animated videos were available at the website and the fourth prompt announced a game on non-smoking.

## Discussion

### Principal Findings

The aims of the present study were to investigate whether prompt messages (via email and SMS) were effective in stimulating primary school children to reuse an intervention website containing information on non-smoking, to assess whether the prompt content was associated with the reuse of the intervention website, and whether there were differences in characteristics between children who responded or did not respond to the prompt messages. Results indicated that prompts had a positive effect on reuse of the intervention website; in particular, prompts that announced new animated movies or games increased reuse more than prompts that announced new information on the website. Additionally, children with a low SES seemed to be even more responsive to the prompts than children with a high SES.

This was the first study on the effects of prompts via email and SMS on reuse of a smoking prevention website among children. Even though the prompt messages seemed to improve website reuse, the total website reuse was still very low, though comparable with what has been found for adults and adolescents [21-23,40]. This low reuse may be explained by the topic (smoking prevention) of the current study. Smoking prevalence rates among Dutch children are low (0% is a daily smoker at age 12) [1] and attitudes of children toward smoking are generally negative [41]. Other explanations may be that children were not interested or thought they had no reason to reuse the intervention website. Besides the topic of the present intervention study, there is, however, room for improvement regarding how to use prompts. One of the solutions may be found in optimizing the frequency of the prompt messages. In this study, we used 6 prompt messages that were sent at different time intervals (1 month or 2 months). Until now, it has only been known that Internet-delivered interventions benefit most from relatively short prompt timing (eg, 2 weeks) [28]; however, it was not known which time periods are most effective. Our goal was to regularly prompt children to reuse the intervention website, but not to overload them with prompt messages. In the development process of the current study, children indicated 1 or 2 months being most appreciated to receive prompt messages. The prompt messages improved reuse of the intervention website, even after the sixth prompt, which was sent almost 1 year after the initial exposure to the intervention. This may demonstrate that prompting children to reuse a website can be effective even over a longer period with changing time intervals. However, it is recommended that future studies put effort in studying the desired frequency of prompt messages by the target group, to maximize the effectiveness of prompts and reuse of the Web-based intervention. Perhaps participants would also be more motivated to respond to prompts if those prompt messages were not imposed on them but instead based on their personal preferences (ie, what kind of prompt messages they prefer to receive and when they prefer to receive them). Another solution to increase website reuse may lie in the content of the prompts. Until now, the evidence toward optimal prompt content has still been lacking [28]. According to the Elaboration Likelihood Model [42], people who are less involved in an intervention are less likely to process information. By tailoring arguments in a persuasive message (ie, prompts), peripheral cues are able to stimulate people to respond to those messages. Armstrong and colleagues [43] showed that prompts were effective in improving adherence when prompt messages contained information that was interesting to the participants, made them curious, or was customized to their personal preferences. Furthermore, interest of participants has shown to be valuable in explaining intervention use [44], since participants with increased interest spent more time reading information on a specific topic [45]. Findings of the present study show that prompt messages containing information on new animated movies and new games stimulated children most to reuse the intervention website, which indicates those messages being most effective. This might be explained since Dutch primary school children are known to be interested in playing online games (68%) or watching short movies on YouTube (95%) [34]. However, it remains unclear whether the presence of new games or animated videos stimulated the children to reuse the intervention website or whether it was the variety in prompt content. This topic should be further investigated in coming studies.

The effects of prompts appeared promising for low SES children. This is especially relevant for them since they suffer more often from health problems than high SES groups [46], are more difficult to reach to participate in Web-based interventions [27,47], and, if they are included in the intervention, they more often refrain from continued use [21,40]. The reason that low SES children seemed to respond a little bit more to the prompts may be that they are more interested in playing online games and watching online videos, whereas high SES children use the Internet more often to search for general information or for school purposes [48]. Another possibility is that low SES children are more curious about smoking, since they engage more often in smoking than high SES children when they make the transition to secondary school [1]. This possibility is also supported by a study of Crutzen et al [49], where adolescents with higher smoking and alcohol drinking intentions were more willing to use an Internet-delivered lifestyle intervention. However, according to our findings, differences concerning reuse between low SES and high SES children are small.

### Strengths and Limitations

A major strength of the present study is the large and diverse sample, since a representative sample of grade 7 children from all regions in the Netherlands was included. The majority of previous studies conducted observational research or lab studies [25] in which no firm conclusions could be given regarding the effectiveness of intervention characteristics or their impact in real life. A further strength was the aggregation of both the data from a Web-based questionnaire with the data regarding use of an intervention website. By using this unique approach, it was possible to associate the individual characteristics of the participants (ie, age, gender, ethnicity, and SES) with the objectively tracked data of the intervention website and, thus, gain more insight into effects of prompts. Despite these strengths, this study was also subject to some limitations. First, it is to be expected that the results presented in this study are less generalizable to countries with less access to technologies such as mobile phones and the Internet. For children in more developed countries, however, these results seem to be promising. Based on national reports [35], it was expected that 60-69% of Dutch primary school children own a mobile phone. However, in the present study, only 15.57% indicated having a mobile phone and even fewer children reported actually using this device. Reasons for these low numbers may be that the children were too young to carry a mobile phone and received one just before they made the transition to secondary school. One advantage of the present study is that prompts were also sent via email, therefore children were still able to receive the prompt messages. Further, we only analyzed if children reused the intervention website but we did not verify whether they visited the website components the prompt message referred to. Our main goal was to test whether prompt messages motivated children to reuse the intervention website, so that they could be exposed to any form of smoking prevention information that was provided on the website. Another limitation might be that we did not verify the email addresses and mobile phone numbers of the children. Too many actions would have had to be taken by the children if verification of the email addresses and mobile phone numbers were mandatory and that may be a reason that they discontinued their participation in the smoking prevention intervention study. However, a tool was developed to correct misspellings in the email addresses or mobile phone numbers, which increased the likelihood that prompts were received correctly by the participating children. A final limitation is that we were not able to objectively assess whether the prompt messages were read by the participating children or if children provided social desirable answers concerning the use of their email or mobile phone. Yet the current study found effects on the number of clicks on the intervention website, which may assure that participants opened and read the prompt messages. However, it remains advisable for future research to put effort in assessing the extent to which prompt messages are actually read and used by the participants to increase reliability of the data.

### Conclusions

Prompt messages via email and SMS can improve reuse of an intervention website with information on smoking prevention among children. Specifically, prompt messages that announced animated videos and games concerning non-smoking stimulated children most to reuse the website. Furthermore, prompt messages seemed to stimulate children of low SES slightly more than high SES children to reuse the intervention website.

## Multimedia Appendix 1

CONSORT-EHEALTH checklist V1.6.2 [50].

## Abbreviations

ANOVA: analysis of variance
SES: socioeconomic status
SMS: short message service
