# Supplementary material for: Do Email and Mobile Phone Prompts Stimulate Primary School Children to Reuse an Internet-Delivered Smoking Prevention Intervention?
Source: J Med Internet Res. 2014 Mar 18;16(3):e86. doi: 10.2196/jmir.3069 (PMC3978553; doi:10.2196/jmir.3069)
Supplement: Supplementary file 1 [file jmir_v16i3e86_app1.pdf]

CONSORT-EHEALTH Checklist V1.6.2 Report

(based on CONSORT-EHEALTH V1.6), available at [http://tinyurl.com/consort-ehealth-v1-6].

3069

Date completed

10/31/2013 7:36:18

by

Henricus-Paul Cremers

Do Email and SMS Prompts Stimulate Primary School Children to Reuse an Internet-delivered Smoking Prevention Website?

TITLE

1a-i) Identify the mode of delivery in the title

"Internet-delivered"

1a-ii) Non-web-based components or important co-interventions in title

1a-iii) Primary condition or target group in the title

"Primary School Children"

ABSTRACT

1b-i) Key features/functionalities/components of the intervention and comparator in the METHODS section of the ABSTRACT

"The sample of this cluster-randomized study consisted of 1,124 children (aged 10 – 11 years) of 108 Dutch primary schools who were assigned to the experimental group of an Internet-delivered smoking prevention intervention study. All participants completed a web-based questionnaire concerning factors related to (non-)smoking. Schools were randomized to a no prompt group (N= 50) or a prompt group (N= 58). All children could revisit the intervention website, but only the children in the prompt group received email and SMS prompts to revisit the website. Those prompt messages functioned as a teaser to stimulate reuse of the intervention website. Reuse of the website was objectively tracked by means of a server registration system. Repeated measures analysis of variance and linear regression analysis were performed to assess the effects of prompts on website reuse and to identify individual characteristics of participants who reuse the intervention website."

1b-ii) Level of human involvement in the METHODS section of the ABSTRACT

1b-iii) Open vs. closed, web-based (self-assessment) vs. face-to-face assessments in the METHODS section of the ABSTRACT

1b-iv) RESULTS section in abstract must contain use data

1b-v) CONCLUSIONS/DISCUSSION in abstract for negative trials

INTRODUCTION

2a-i) Problem and the type of system/solution

"Evidence on how children respond to prompt messages when they are involved in an Internet-delivered intervention is scarce. Therefore, it is important to study the effects of prompts in primary school children to increase the potential reuse of (effective) interventions."

2a-ii) Scientific background, rationale: What is known about the (type of) system

"Periodic prompts may be a valuable tool to stimulate reuse of an Internet-delivered intervention. Previous studies among adults and adolescents already demonstrated that the provision of (periodic) prompts had a positive effect on reuse of an intervention website, for example on the number of log-ins."

"Prior research among adults has indicated that participants were more willing to log-in to an intervention website if they received a prompt messages containing a preview of new information compared to standard prompt messages (a message that reminded people of their previous visit and invited them to reuse the website without addressing the new content added to the intervention website). Furthermore, it is plausible that individual characteristics of participants, such as age, gender or socioeconomic status (SES), are associated with whether or not to reuse an Internet-delivered intervention."

METHODS

3a) CONSORT: Description of trial design (such as parallel, factorial) including allocation ratio

"The objectives of the present study are to examine 1) whether prompts will stimulate primary school children to reuse a smoking prevention website; 2) whether the prompt content is related to its effect in terms of reuse and 3) which individual characteristics of children are associated with a higher likelihood to respond to the prompts and reuse of an intervention website."

3b) CONSORT: Important changes to methods after trial commencement (such as eligibility criteria), with reasons

Not applicable

3b-i) Bug fixes, Downtimes, Content Changes

4a) CONSORT: Eligibility criteria for participants

"Since prompts were sent via email and SMS, inclusion criteria for the present study were that children had entered a proper email address or mobile phone number and that they had indicated to actually use this email address or mobile phone number."

4a-i) Computer / Internet literacy

4a-ii) Open vs. closed, web-based vs. face-to-face assessments:

"Participants in the present study are children in grade 7 (aged 10 – 11 years). Primary schools were recruited by Municipal Health Promotion Organizations and Maastricht University for participation in the smoking prevention intervention study. Children in grade 7 of all participating schools were included in the intervention study, unless they or their parents refused to be involved (passive informed consent procedure)."

4a-iii) Information giving during recruitment

4b) CONSORT: Settings and locations where the data were collected

"In October 2011, all children received personalized log-in codes (username and password) to access the 'Fun without Smokes' website (figure 1) and were asked to fill-out a web-based questionnaire at their primary school concerning their smoking status and other factors related to smoking."

4b-i) Report if outcomes were (self-)assessed through online questionnaires

"In October 2011, all children received personalized log-in codes (username and password) to access the ‘Fun without Smokes’ website (figure 1) and were asked to fill-out a web-based questionnaire at their primary school concerning their smoking status and other factors related to smoking."

"Primary outcome measure of the present study is reuse of the ‘Fun without Smokes’ website. Use and reuse of the website was assessed objectively by means of a server registration system."

4b-ii) Report how institutional affiliations are displayed

5) CONSORT: Describe the interventions for each group with sufficient details to allow replication, including how and when they were actually administered

5-i) Mention names, credential, affiliations of the developers, sponsors, and owners

5-ii) Describe the history/development process

5-iii) Revisions and updating

5-iv) Quality assurance methods

5-v) Ensure replicability by publishing the source code, and/or providing screenshots/screen-capture video, and/or providing flowcharts of the algorithms used

5-vi) Digital preservation

5-vii) Access

"In October 2011, all children received personalized log-in codes (username and password) to access the ‘Fun without Smokes’ website (figure 1) and were asked to fill-out a web-based questionnaire at their primary school concerning their smoking status and other factors related to smoking."

"The ‘Fun without Smokes’ website was accessible for both the children in the prompt and no prompt group during the intervention period. Core elements of the ‘Fun without Smokes’ website were the web-based questionnaire and the computer-tailored feedback letters. Furthermore, the website provided information on non-smoking through facts concerning non-smoking, anti-smoking games and short animated videos with non-smoking content."

"Participants in the no prompt group had also access to the renewed information, games and videos. However, reuse of the ‘Fun without Smokes’ website was dependent on their own initiative since they did not receive any of the 6 prompt messages. They received their personal log-in codes at the baseline measurement of ‘Fun without Smokes’ and were asked to save those codes. If they had lost the codes, they were able to request them at the ‘Fun without Smokes’ website."

5-viii) Mode of delivery, features/functionalities/components of the intervention and comparator, and the theoretical framework

"In October 2011, all children received personalized log-in codes (username and password) to access the ‘Fun without Smokes’ website (figure 1) and were asked to fill-out a web-based questionnaire at their primary school concerning their smoking status and other factors related to smoking. After completion children in both the prompt and no prompt group received 3 personalized computer-tailored feedback letters in their own email box and at the ‘Fun without Smokes’ website. Those feedback letters were not only tailored to children’s personalities, but also to socio-cognitive variables (e.g. attitude, social influences and self-efficacy expectations) toward (non-)smoking. The children in the prompt group received 6 prompt messages to stimulate them to reuse the ‘Fun without Smokes’ website, where they could read new information concerning (non-)smoking, play games or watch animated videos with non-smoking content. After children completed the questionnaire at school, they were able to use the web-based intervention at home."

"In the computer-tailored feedback letters it was indicated that participants in both the prompt and no prompt group were able to reuse the website during the intervention period. Children who had entered an email and/or mobile phone number in the prompt group received 6 prompt messages within 9 months to stimulate them to reuse the ‘Fun without Smokes’ website. The prompts were sent via email and/or as SMS messages, depending on whether the child had provided an email account and/or mobile phone number and had indicated to use this device. Children received an email and SMS message if they provided both (i.e. email address and phone number), otherwise they received only an email or SMS. Children without email or mobile phone did not receive the prompts."

5-ix) Describe use parameters

5-x) Clarify the level of human involvement

5-xi) Report any prompts/reminders used

"In the computer-tailored feedback letters it was indicated that participants in both the prompt and no prompt group were able to reuse the website during the intervention period. Children who had entered an email and/or mobile phone number in the prompt group received 6 prompt messages within 9 months to stimulate them to reuse the ‘Fun without Smokes’ website. The prompts were sent via email and/or as SMS messages, depending on whether the child had provided an email account and/or mobile phone number and had indicated to use this device. Children received an email and SMS message if they provided both (i.e. email address and phone number), otherwise they received only an email or SMS. Children without email or mobile phone did not receive the prompts.
All prompt messages varied in content and were sent in different time periods. The first 3 prompts were sent 1, 2 and 3 months after the baseline questionnaire was completed. The last 3 prompts were sent 5, 7 and 9 months after baseline. In accordance with the prompts, some of the content of the intervention website was refreshed to address a new topic relevant for smoking prevention. The prompts functioned as a teaser to increase curiosity among the children to view the new content at the ‘Fun without Smokes’ website (e.g. Hi, now there is a funny game on the Fun without Smokes website. Check it out today and play this game!). The content of the first and second prompt notified that new facts on (non-)smoking were posted at the website (table 1), the third prompt announced that new animation videos (including non-smoking messages) were posted at the website, the fourth prompt reported that a game was available about (non-)smoking, the fifth prompt mentioned new facts about (non-)smoking and the last prompt announced a new game of non-smoking. Every prompt also included the personal log-in codes of the ‘Fun without Smokes’ website, to make sure that the children could access the website immediately.Participants in the no prompt group had also access to the renewed information, games and videos. However, reuse of the ‘Fun without Smokes’ website was dependent on their own initiative since they did not receive any of the 6 prompt messages. They received their personal log-in codes at the baseline measurement of ‘Fun without Smokes’ and were asked to save those codes. If they had lost the codes, they were able to request them at the ‘Fun without Smokes’ website."

5-xii) Describe any co-interventions (incl. training/support)

Not applicable

6a) CONSORT: Completely defined pre-specified primary and secondary outcome measures, including how and when they were assessed
"Primary outcome measure of the present study is reuse of the 'Fun without Smokes' website. Use and reuse of the website was assessed objectively by means of a server registration system. Reuse was measured as a continuous variable, based on the number of clicks (ranging from 0 to 95). Characteristics of the user and reuser such as age, gender ethnicity and SES were derived from the baseline questionnaire that the children completed in the classroom on the 'Fun without Smokes' website."

"The data from the server registration system and the data from the baseline questionnaire could be linked by means of the personal usernames, making it possible to unobtrusively observe if a participant reused the intervention website after a prompt message was sent and to combine usage information with individual data of the users."
6a-i) Online questionnaires: describe if they were validated for online use and apply CHERRIES items to describe how the questionnaires were designed/deployed

6a-ii) Describe whether and how "use" (including intensity of use/dosage) was defined/measured/monitored

6a-iii) Describe whether, how, and when qualitative feedback from participants was obtained

6b) CONSORT: Any changes to trial outcomes after the trial commenced, with reasons
Not applicable
7a) CONSORT: How sample size was determined
7a-i) Describe whether and how expected attrition was taken into account when calculating the sample size

7b) CONSORT: When applicable, explanation of any interim analyses and stopping guidelines
Not applicable
8a) CONSORT: Method used to generate the random allocation sequence
"The study was conducted as a cluster-randomized controlled trial in which 108 primary schools in the Netherlands were randomized to either a prompt (N= 58) or no prompt group (N= 50) of a larger smoking prevention intervention study, called 'Fun without Smokes'."
8b) CONSORT: Type of randomisation; details of any restriction (such as blocking and block size)
"cluster-randomized controlled trial"
9) CONSORT: Mechanism used to implement the random allocation sequence (such as sequentially numbered containers), describing any steps taken to conceal the sequence until interventions were assigned
Randomisation is carried out by a computer-program. Mechanism is not described in the present manuscript, since it was not relevant for the present study.
10) CONSORT: Who generated the random allocation sequence, who enrolled participants, and who assigned participants to interventions
Randomisation is performed at school level and carried out by a computer-program. Mechanism is not described in the present manuscript, since it was not relevant for the present study
11a) CONSORT: Blinding - If done, who was blinded after assignment to interventions (for example, participants, care providers, those assessing outcomes) and how
11a-i) Specify who was blinded, and who wasn't
Not applicable
11a-ii) Discuss e.g., whether participants knew which intervention was the "intervention of interest" and which one was the "comparator"

11b) CONSORT: If relevant, description of the similarity of interventions
Not relevant
12a) CONSORT: Statistical methods used to compare groups for primary and secondary outcomes
"General descriptives were carried out to describe the sample under study. Differences at baseline between characteristics of children (i.e. age, gender, ethnicity, SES, having/using their email address and having/using their mobile phone) in the no prompt and prompt group were analyzed with chi-square (X2) and t-test analyses (t).
A multiple linear regression analysis was conducted to identify whether there was a difference in website reuse between the prompt and the no prompt group. In this analysis reuse of the intervention website was the dependent variable and group and demographic characteristics the independent variables. To identify whether there were differential effects of the prompt condition based on demographic characteristics a linear regression analysis was done that included group*demographic variable interaction terms (i.e. age, gender, ethnicity or SES). If interaction effects were present, separate analyses were performed for two subgroups of a variable.
To indicate which prompt(s) motivated children most to reuse the 'Fun without Smokes' website a repeated-measures analysis of variance (ANOVA) was carried out. In this analysis the number of clicks in the separate prompt periods were analyzed between the prompt and no prompt group. All analyses were performed in SPSS 20.0. P-values were said to be significant if they were equal to or lower than .05. Interaction effects were considered to be significant if the P-value was equal to or lower than .10 to reduce potential type I errors."
12a-i) Imputation techniques to deal with attrition / missing values
Not relevant in the present study. If people did not use the intervention anymore, did not automatically mean that they dropped out.
12b) CONSORT: Methods for additional analyses, such as subgroup analyses and adjusted analyses
"To identify whether there were differential effects of the prompt condition based on demographic characteristics a linear regression analysis was done that included group*demographic variable interaction terms (i.e. age, gender, ethnicity or SES). If interaction effects were present, separate analyses were performed for two subgroups of a variable."

RESULTS

13a) CONSORT: For each group, the numbers of participants who were randomly assigned, received intended treatment, and were analysed for the primary outcome
"A total of 1,124 children met the inclusion criteria and were included in the analyses (13.9% was excluded)."

In the prompt group: 586 children
In the no prompt group: 538 children
13b) CONSORT: For each group, losses and exclusions after randomisation, together with reasons
Not applicable for the present study
13b-i) Attrition diagram

14a) CONSORT: Dates defining the periods of recruitment and follow-up

"Data gathered during the first year of the intervention study (October 2011 – September 2012) was used in the analyses."

14a-i) Indicate if critical “secular events” fell into the study period

14b) CONSORT: Why the trial ended or was stopped (early)

Only the data during the first year are presented. The intervention is still going on.

15) CONSORT: A table showing baseline demographic and clinical characteristics for each group

"As shown in table 2, in the total sample more girls (55.4%) and more children of a Western ethnic background were included (85.5%). Furthermore, fewer children were of high SES (43.1%). None of the differences between the prompt and the no prompt group were statistically significant. Most children had an email address and made use of this email address (> 98%). A minority of the children had and used a mobile phone (< 16%)."

15-i) Report demographics associated with digital divide issues

In table 2 the following demographic are mentioned: age, gender, ethnicity, SES, having a email address, using email, having a mobile phone, using mobile phone.

16a) CONSORT: For each group, number of participants (denominator) included in each analysis and whether the analysis was by original assigned groups

16-i) Report multiple “denominators” and provide definitions

"Mean reuse of the intervention website was 2.14 times (SD= 7.53) in the prompt group and 0.47 times (SD= 2.30) in the no prompt group and this difference was significant (B= 1.56 (P< .001))."

16-ii) Primary analysis should be intent-to-treat

17a) CONSORT: For each primary and secondary outcome, results for each group, and the estimated effect size and its precision (such as 95% confidence interval)

"Mean reuse of the intervention website was 2.14 times (SD= 7.53) in the prompt group and 0.47 times (SD= 2.30) in the no prompt group and this difference was significant (B= 1.56 (P< .001))."

17a-i) Presentation of process outcomes such as metrics of use and intensity of use

17b) CONSORT: For binary outcomes, presentation of both absolute and relative effect sizes is recommended

Not applicable

18) CONSORT: Results of any other analyses performed, including subgroup analyses and adjusted analyses, distinguishing pre-specified from exploratory

"Mean reuse of the intervention website in the prompt group among children of low SES was 3.03 times (SD= 9.84) and among high SES children 1.37 times (SD= 4.62). Moreover, table 3 shows that only the ‘group by SES’ interaction term was significant (B= -1.22 (P= .06)). Analyses stratified for high and low SES revealed that children of low SES in the prompt group used the website more often (B= 2.19 (P< .001)) than high SES children in de prompt group (B= 0.93 (P= .005)). There was no significant difference observed of SES in the no prompt group."

"In figure 2 the mean reuse of the website at all 6 time points is plotted for the prompt and no prompt group. Reuse of the website is higher in the prompt group as compared to the no prompt group after every prompt (F1, 1122 = 3.66, P= .04), with larger differences between the second and third (F1, 1122 = 9.33, P= .002) and between the third and fourth prompt period (F1, 1122 = 8.28, P= .004). The third prompt announced that new animated videos were available at the website and the fourth prompt announced a game on non-smoking."

18-i) Subgroup analysis of comparing only users

19) CONSORT: All important harms or unintended effects in each group

Not applicable

19-i) Include privacy breaches, technical problems

19-ii) Include qualitative feedback from participants or observations from staff/researchers

DISCUSSION

20) CONSORT: Trial limitations, addressing sources of potential bias, imprecision, multiplicity of analyses

20-i) Typical limitations in ehealth trials

"First, we only analyzed if children reused the intervention website but did not verify if they visited the website components where the prompt message referred to. Our main goal was to test if prompt messages motivated children to reuse the intervention website, so that they could be exposed to any form of smoking prevention information that was provided on the website. Another limitation might be that we did not verify the email addresses and mobile phone numbers of the children. Too many actions had to be taken by the children if a verification of the email addresses and mobile phone numbers was mandatory and that may be a reason that they discontinued their participation in the smoking prevention intervention study. However, a tool was developed to correct misspellings in the email addresses or mobile phone numbers, which increases the likelihood that prompts were received correctly by the participating children. A final limitation is that we were not able to objectively assess if the prompt messages were read by the participating children. Though, the current study found effects on the number of clicks on the intervention website, which may indicate that participants read the prompt messages. However, it remains advisable for future research to put effort in assessing the degree to which prompt messages are actually read by the participants to increase reliability of the data."

21) CONSORT: Generalisability (external validity, applicability) of the trial findings

21-i) Generalizability to other populations

21-ii) Discuss if there were elements in the RCT that would be different in a routine application setting

22) CONSORT: Interpretation consistent with results, balancing benefits and harms, and considering other relevant evidence

22-i) Restate study questions and summarize the answers suggested by the data, starting with primary outcomes and process outcomes (use)

"The aims of the present study were to investigate whether prompt messages (via email and SMS) were effective in stimulating primary school children to reuse an intervention website containing information on non-smoking, to assess whether the prompt content was associated with the reuse of the intervention website and whether there were differences in characteristics between children who responded or did not respond to the prompt messages. Results indicated that prompts had a positive effect on reuse of the intervention website, especially prompts that announced new animation movies or games increased reuse more than prompts that announced new information on the website. Additionally, children with a low SES seemed to be even more responsive to the prompts than children with a high SES."

22-ii) Highlight unanswered new questions, suggest future research

Other information

23) CONSORT: Registration number and name of trial registry

"This study was approved by the Medical Ethics Committee of the Atrium-Orbis-Zuyd Hospital (NL32093.096.11/MEC 11-T-25) and registered in the Dutch Trial Register (NTR3116)."

24) CONSORT: Where the full trial protocol can be accessed, if available

Cremers HP, Mercken L, Oenema A, de Vries H. A web-based computer-tailored smoking prevention programme for primary school children: intervention design and study protocol. BMC Public Health 2012;12:277. doi: 10.1186/1471-2458-12-277 [PMID:22490110]

http://www.biomedcentral.com/1471-2458/12/277

25) CONSORT: Sources of funding and other support (such as supply of drugs), role of funders

"This work was supported by ZonMw, the Netherlands Organization for Health Research and Development [200110011]."

X26-i) Comment on ethics committee approval

x26-ii) Outline informed consent procedures

X26-iii) Safety and security procedures

X27-i) State the relation of the study team towards the system being evaluated
